# The Sensing Liver: Localization and Ligands for Hepatic Murine Olfactory and Taste Receptors

**DOI:** 10.3389/fphys.2020.574082

**Published:** 2020-10-06

**Authors:** Ryan Kurtz, Lily G. Steinberg, Madison Betcher, Dalton Fowler, Blythe D. Shepard

**Affiliations:** Department of Human Science, Georgetown University, Washington, DC, United States

**Keywords:** olfactory receptors, taste receptors, liver, terpene, pyrazine

## Abstract

Sensory receptors, including olfactory receptors (ORs), taste receptors (TRs), and opsins (Opns) have recently been found in a variety of non-sensory tissues where they have distinct physiological functions. As G protein-coupled receptors (GPCRs), these proteins can serve as important chemosensors by sensing and interpreting chemical cues in the environment. We reasoned that the liver, the largest metabolic organ in the body, is primed to take advantage of some of these sensory receptors in order to sense and regulate blood content and metabolism. In this study, we report the expression of novel hepatic sensory receptors – including 7 ORs, 6 bitter TRs, and 1 Opn – identified through a systematic molecular biology screening approach. We further determined that several of these receptors are expressed within hepatocytes, the parenchymal cells of the liver. Finally, we uncovered several agonists of the previously orphaned hepatic ORs. These compounds fall under two classes: methylpyrazines and monoterpenes. In particular, the latter chemicals are plant and fungal-derived compounds with known hepatic protective effects. Collectively, this study sheds light on the chemosensory functions of the liver and unveils potentially important regulators of hepatic homeostasis.

## Introduction

Sensory receptors including olfactory (ORs) and taste receptors (TRs), and photo opsins (Opns) are 7-transmembrane domain G protein-coupled receptors (GPCRs) that have well known functions in the olfactory epithelium, taste buds, and eyes, respectively. Recently, these proteins have also been shown to have important roles in other tissues as well. For example, ectopically expressed ORs have been found throughout the body including the testis (Feldmesser et al., 2006; Kang and Koo, 2012), heart (Feldmesser et al., 2006; Kang and Koo, 2012; Flegel et al., 2013), kidney (Feldmesser et al., 2006; Kang and Koo, 2012; Pluznick and Caplan, 2012; Flegel et al., 2013; Rajkumar et al., 2014; Halperin Kuhns et al., 2019), brain (Feldmesser et al., 2006; Kang and Koo, 2012; Flegel et al., 2013), thyroid (Feldmesser et al., 2006; Flegel et al., 2013), and pancreas (Feldmesser et al., 2006; Kang and Koo, 2012), while TRs and Opns have also been identified in the airway epithelium (Li, 2013; Gilca and Dragos, 2017), large and small intestine (Li, 2013; Gilca and Dragos, 2017), adrenal glands (Halford et al., 2001; Li, 2013), and kidney (Halford et al., 2001). While the physiological relevance of these receptors is continuing to emerge, many of these ‘extra sensory’ localizations have been linked to physiological and pathophysiological functions (Spehr et al., 2003; Neuhaus et al., 2009; Sharma et al., 2017; Abaffy et al., 2018; Massberg and Hatt, 2018; Lee et al., 2019; Tole et al., 2019). Work from our laboratory has focused on the characterization of renal OR Olfr1393 and its role in glucose handling (Shepard et al., 2016, 2019) and diabetes (Shepard et al., 2019). ORs have also been shown to function in the regulation of adiposity, blood pressure, and carcinoma proliferation (Pluznick et al., 2013; Massberg et al., 2015; Wu et al., 2015). TRs have been implicated in glucose and lipid metabolism (Halford et al., 2001; Wu S. et al., 2019) as well as bronchodilation (Deshpande et al., 2010) and Opns have proposed extraocular functions such as rhythmic clock-gene expression and sperm thermotaxis (Leung and Montell, 2017).

The liver is the largest metabolic organ, and it is tasked with regulating whole-body homeostasis by sensing and detoxifying xenobiotics, producing and metabolizing glucose, synthesizing and secreting bile acids, and removing bacteria from the blood. Therefore, the liver appears to be primed to take advantage of these sensory GPCRs. Indeed, several metabolites, including intermediates of the citric acid cycle have been shown to target GPCRs in the gut and liver to modulate metabolism, lending credence to the potential for hepatic sensory receptors to have pertinent physiological functions (He et al., 2004). In addition, major urinary proteins (MUPs), which are secreted by the liver and excreted into the urine, bind to volatile chemicals to regulate their circulation and trigger adaptive physiological responses. In particular, MUP1 has been shown to regulate glucose and lipid metabolism further supporting the notion that volatile compounds (many of which are detected by ORs) can elicit physiological action (Zhou and Rui, 2010). In particular, cholangiocytes are a likely candidate for functional OR expression, as the cilia of these epithelial cells are already known to have a chemosensory role (Mansini et al., 2019).

Recently, several groups have identified functions for hepatic ORs. For example, Olfr734 responds to the hormone Asprosin, which triggers glucose production via gluconeogenesis in the liver (Li et al., 2019). Olfr544, which has been found to be highly expressed in both liver and adipose tissue, can trigger lipolysis upon activation in diabetic mice (Wu et al., 2017). Finally, human ORs, OR10J5 and OR1A1, have been found in liver hepatoma cell lines and appear to contribute to triglyceride metabolism (Wu et al., 2015; Tong et al., 2017).

While there have been individual reports of ectopically-expressed sensory receptors, there has yet to be a more wide-scale screen for ORs, TRs, and Opns in the liver. Here we describe the systematic identification and characterization of murine hepatic sensory receptors. Using custom-made TaqMan arrays and a confirmatory molecular biology approach, we have identified a total of 30 sensory receptors expressed in the murine liver. Further analysis of the highest expressing ORs and TRs has revealed a hepatic localization and the deorphanization of two ORs. Ligand profiles reveal that one of these receptors responds to monoterpenes, plant-based metabolites with known hepatic protective effects, while the other responds to methylated pyrazines, an under-researched class of odorants. Collectively, this data expands on the known “sensory” functions of the liver and sheds light into the physiological relevance of these newly identified extra nasal ORs.

## Materials and Methods

### Liver Harvesting and RNA Isolation

C57Bl/6 male and female mice were euthanized by CO_2_ asphyxiation and livers were quickly collected and flash frozen in liquid nitrogen. Tissues were homogenized in Trizol (Invitrogen) and RNA was isolated using a phenol-chloroform extraction followed by a DNaseI (Invitrogen) digest. All animal experiments were approved and performed in accordance with the policies and procedures of the Georgetown University Institutional Animal Care and Use Committee.

### Taqman Array Screen

To identify novel GPCRs and determine their relative expression levels in the liver, we performed a screen of whole liver tissue cDNA using a custom sensory receptor TaqMan array card according to the manufacturers protocol (Thermo Fisher). This array was designed to screen for the complete complement of bitter, sweet, and umami TRs, all non-visual Opns, and 44 ORs that are highly likely to be ectopically expressed. The complete list of sensory receptors included in the array is listed in Supplementary Table 1. Briefly, RNA was isolated from male C57BL6 livers using phenol-chloroform extraction and 2 μg of RNA was used to synthesize cDNA using the High Capacity RNA-to-cDNA Kit (Applied Biosystems). Each reservoir in the given Taqman array microfluidic card was filled with 1,000 ng of cDNA and the array cards were run on the 7900 HT- RT-PCR system (Applied Biosystems) and analyzed using the SDS software. Each gene was run in triplicate and Δ*C*t values for each receptor was calculated using 18s ribosomal RNA. The initial screen was intended to identify the complete complement of sensory receptors expressed in murine liver and as such, standard deviation was not determined (*n* = 2 livers).

### RT-PCR

All sensory receptors identified from the TaqMan array screen were confirmed by performing endpoint PCR on liver cDNA from both male and female livers (*N* = 4–6) using both gene-specific and full-length primer sets (nucleotide sequences and expected product size listed in Supplementary Tables 2, 3). For use with gene-specific screening primers (GSPs), tissue-specific cDNA was synthesized from 1 μg of purified RNA by reverse transcription (RT; iScript cDNA Synthesis Kit, Bio-Rad). Mock-reverse transcription controls were also prepared alongside using an equal volume of water in lieu of reverse transcriptase. PCR screening with GSPs was performed using HotStarTaq Master Mix (Qiagen) following standard thermocycling conditions. Given that these sensory receptors are single-exon coding genes, cycling conditions were first optimized using genomic DNA. Mock RT reactions were run in parallel with all RT reactions, and all PCR amplicons were sequenced to confirm identity.

To isolate full-length PCR products, tissue-specific cDNA was synthesized from 2 μg of purified RNA by reverse transcription using a High Capacity RNA-to-cDNA Kit (Applied Biosystems) and full-length primers were used to amplify the highest expressing receptors using either Q5 2X Master Mix (NEB), or Phusion High-Fidelity Polymerase (NEB). In order to optimize PCR conditions for difficult ORs, a Touch-down PCR protocol was used as detailed previously (Korbie and Mattick, 2008). All full-length products were confirmed by sequencing and cloned into a pME18s vector containing a cleavable Lucy tag (Shepard et al., 2013) and a Rho tag (Krautwurst et al., 1998) to aid in receptor trafficking to the cell membrane.

### Surface Immunofluorescence

HEK293T cells were seeded onto 18-mm coverslips coated with poly-L-lysine and transiently transfected with OR constructs with or without accessory proteins RTP1s and Ric8b (Lipofectamine 2000, Invitrogen). Flag-tagged OR trafficking was assayed using surface immunofluorescence as previously described (Shepard et al., 2013). Briefly, live, non-permeabilized cells at 4°C were exposed to a rabbit polyclonal anti-Flag antibody (Sigma) in 0.1% BSA/PBS. This antibody will only detect the extracellular Flag epitope of those receptors that are functionally expressed on the plasma membrane. Subsequently, cells were washed, fixed with 4% paraformaldehyde, permeabilized (0.3% Triton X-100), and then exposed to a mouse monoclonal (M2) anti-Flag antibody (Sigma). As the external Flag epitope (surface Flag) is ‘blocked’ after binding to the polyclonal Flag antibody, the monoclonal Flag antibody detects only the internal population of ORs. Alexa Fluor fluorescent secondary antibodies (Thermo Fisher) were used to detect the localization of the polyclonal and monoclonal Flag-tags. Slides were imaged using the ZEISS Axiovert 200 m microscope at 40× magnification.

### cAMP-Dependent Dual Luciferase Assay

For ORs whose trafficking conditions could be established as described above, an unbiased ligand screen using a dual-luciferase reporter assay (Promega) was used to identify potential ligands. Briefly, ORs were transiently transfected (Lipofectamine 2000, Invitrogen) in triplicate into HEK293T cells along with constructs encoding for CREB-dependent luciferase (Firefly) and a constitutively expressed luciferase (Renilla), along with any accessory proteins previously established to be required for effective cell-surface trafficking. Additional surface labeling experiments were performed with the Firefly and Renilla constructs to ensure effective trafficking was not lost under these transfection conditions. OR activation leads to a rise in cAMP which drives an increase in Firefly luciferase expression. Firefly activity is normalized to the activity of the Renilla luciferase to control for variation in cell number and transfection efficiency. Data were collected using a FLUOstar Omega automated plate reader (BMG LabTech, Cary, NC, United States). Cells were allowed to incubate for at least 30 min in CD293 minimal media, and were then bathed with potential odorants diluted in minimal media for 4 h before measuring Firefly and Renilla-derived luminescence. Initially, screening was performed using odorant mixes that are grouped mainly by functional groups (BzB: nonanal, heptanal, valeraldehyde; BzC: carvone, eugenol, cinnamaldehyde; MA: amyl acetate, 3-octanone, acetophenone; OXLK: 2,3-butanedione, pyruvaldehyde, acetic acid, 1,2-ethanedithiol, 2-butanone; THI-DI: 1,6-hexanedithiol, 1,2-ethanedithiol, 2-methyl-1-propanethiol, 1,2-butanedithiol, 2,3-butanedithiol). These mixtures are known to elicit activation of a large number of olfactory sensory neurons and thus cover a wide range of ‘odorant space’ (Pluznick et al., 2013), followed by any known ligands to ORs in the same family as those being tested, as well as physiologically relevant substances. A complete list of chemicals used for screening is found in Supplementary Table 1 and all chemicals tested were obtained from Sigma or Fisher Scientific. To begin, all compounds screened were tested at a minimum of two concentrations (100–300 μm and 1–5 mM) to cover a wide range of potential activation. For each assay, Olfr78 or Olfr1393 and known activators of these receptors (Propionate and Cycloheptanol, respectively) were used as positive controls. In order to determine specific activation, potential activators of one receptor were also tested on at least one other OR simultaneously. Negative controls were performed with both media, and media containing DMSO. When possible, EC_50_ values were calculated using Prism Graphpad software using a dose response of at least 6 concentrations (note: the response curve for 2,3,5-trimethylpyrazine failed to plateau preventing calculation of a reliable EC_50_ value). A significant rise in Firefly:Renilla over baseline was determined by multiple *T*-Tests (*P* > 0.05 deemed significant).

### RNAscope

To localize sensory receptors within the liver, RNAscope was performed using a commercially available probe for Tas2r108 and a custom-designed probe for Olfr57. Both male and female C57BL6 mouse livers (*n* = 4) were obtained following perfusion fixation with 100 ml phosphate buffered 4% paraformaldehyde solution. The livers were collected, post-fixed in 4% PFA for 24 h and were equilibrated in increasing concentrations of sucrose solutions (10–30%) until the tissue sank to the bottom of the solution. Tissues were then embedded into OCT blocks and stored at −80°C. 14 μm sections were cut by cryostat and mounted on SuperFrost Plus slides, dried for 1 h at −20°C, and stored at −80°C until use. RNAscope was performed according to manufacturer’s protocol (Advanced Cell Diagnostics). Briefly, fixed frozen slides were prefixed with a 30-min baking step at 60°C followed by a 15-min post fix in 4% PFA at 4°C. Following hydrogen peroxide treatment, target retrieval was performed for 5 min at 100°C followed by protease treatment for 15 min at 40°C (Protease III solution). Probes were hybridized for 2 h at 40°C and stored overnight in a citrate solution (5X SSC). The following day, RNAscope amplification and washing steps were followed as per the manufacturer’s protocol. Three RNAscope positive control probes (ubiquitin C; HS-UBC, HS-PPIB, and HS-POLR2A) were used on each tissue to assess RNA integrity while probe diluent was used as a negative control. For fluorescent detection, the labeled probe was conjugated to TSA Plus Fluorescein, TSA Plus Cyanine 3, or TSA Cyanine 5 (PerkinElmer). Post treatment slides were counterstained with DAPI, mounted with Vectashield, and allowed to dry for 24 h prior to imaging on a fluorescent ZEISS Axiovert 200 m microscope at 63× magnification. For Olfr57 RNAscope, 5 *Z*-stacks were obtained; images were deconvoluted and a maximum intensity projection of all *Z* stacks were obtained using Zen post-image processing.

Prior to probing the liver, the functionality of both probes was confirmed on overexpressed HEK293T cells. Briefly, HEK293T cells were passaged on to chamber slides (Thermo Fisher) and transfected (Lipofectamine 2000) with Flag-tagged DNA constructs for Tas2r108 or Olfr57. Chamber slides were disassembled and rinsed with PBS. Slides were then fixed at room temperature for 30 min in 10% neutral buffered formalin and dehydrated using an ethanol series. RNAscope and immunofluorescence were then performed as described above.

### Alpha Mouse Liver 12 (AML12) Cells

Murine hepatic AML12 cells (ATCC) were cultured in DMEM:F12 media supplemented with 10% fetal bovine serum, 40 ng/ml dexamethasone, and 1× insulin-transferrin-selenium (ITS) solution (Invitrogen). Cells (passage #3) were grown on 25 cm^2^ flasks until they reached full confluence and RNA was isolated using the RNAeasy Mini Kit (Qiagen) according to manufacturer’s protocol. To identify full-length ORs and TRs expressed in the hepatic cell line, 2 μg of RNA was reverse transcribed to cDNA and full-length primers were used as described above.

## Results

### Sensory Receptors Are Expressed in Murine Liver

Sensory receptors including ORs, TRs, and Opns are known to play important roles in seemingly ‘non-sensory’ tissues. We hypothesized that the liver, the largest metabolic organ in the human body, is primed to take advantage of these receptors in order to sense and regulate its internal environment. To identify the complete complement of these receptors expressed in murine liver, we generated a custom TaqMan array to screen for all bitter, sweet, and umami TRs, the non-visual Opns, and 44 ORs including all of the ORs with known extra-nasal functions (complete TaqMan array found in Supplementary Table 1). In total, we were able to screen for 87 sensory receptors in murine male liver and identified a total of 30 sensory receptors: 17 ORs, 10 TRs, 3 Opns (Table 1).

**TABLE 1:** Sensory receptors expressed in murine liver.

**Sensory receptor**	Δ*C*t*	**Expression confirmed in >4 livers?**	**Full-length product confirmed?**
Olfr99	22.1	+	+
Olfr267	22.5	+	+
Olfr1393	23.5	+	−
Olfr1366	25.4	+	+
Olfr691	25.8	+	−
Olfr558	26.2	+	−
Olfr57	26.3	+	+
Olfr646	26.6	+	−
Olfr78	26.7	+	+
Olfr15	26.9	+	−
Olfr177	26.9	+	+
Olfr308	26.9	+	−
Olfr90	27.1	+	−
Olfr418	27.3	−	−
Olfr545	27.3	+	−
Olfr873	27.4	+	−
Olfr56	27.5	+	−
Tas2r1r3	22.7	+	N/D
Tas2r135	23.5	+	+
Tas2r143	23.7	+	+
Tas2r108	23.8	+	+
Tas2r137	24.1	+	+
Tas2r126	25.2	+	+
Tas1r2	25.7	+	N/D
Tas1r1	25.8	−	
Tas2r138	26.4	+	+
Tas2r106	27.5	−	−
Opn3	22.0	+	+
Opn1sw	24.3	+	−
Opn4	26.7	−	−
Reference gene	**Δ** *C*t*		
Gapdh	10.5		

*ΔCt is defined as the difference in cycling threshold between the genes of interest and 18s rRNA.

**FIGURE 1:**
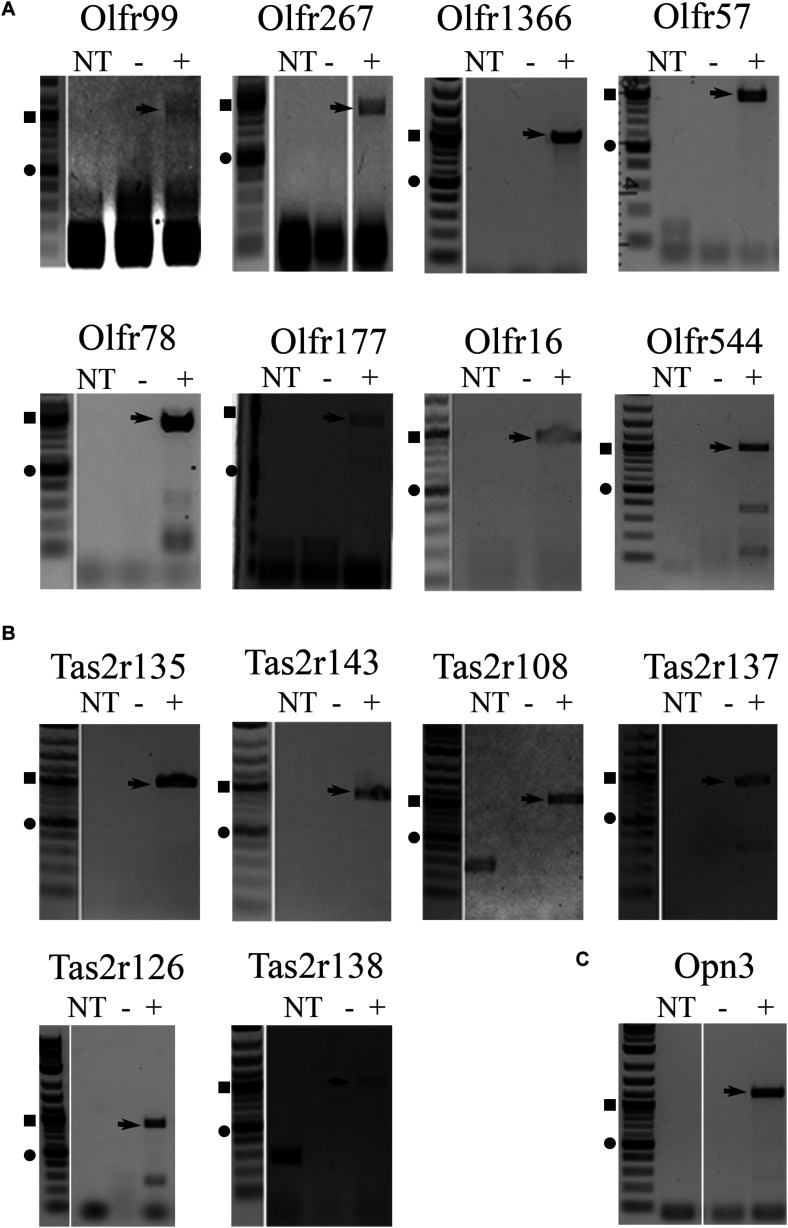
RT-PCR for full-length transcripts of olfactory receptors, taste receptors, and opsins confirm expression of sensory receptors in the liver. RT-PCR on both male and female liver cDNA was performed using full-length primers to confirm expression of **(A)** olfactory receptors, **(B)** taste receptors, and **(C)** opsins identified from the TaqMan array screen (Table 1) and confirmatory PCR (Supplementary Figure 1). Representative, cropped gel images are shown with arrows noting the sequenced product. The DNA ladder is shown to the left of each gel with the square symbol marking 1,000 bp and the circle at 500 bp. NT, no template control, – = mock RT reaction, += RT reaction.

To confirm expression of the identified receptors, RNA isolated from whole liver samples (*n* ≥ 4) was reverse transcribed and end-point PCR was performed using gene-specific primers. The vast majority of these sensory receptors are single-coding exons and thus, both reverse-transcribed (+) and mock (−) reactions were performed simultaneously. Out of the initial array card, we were able to confirm the expression of 26 sensory receptors including 16 ORs, 8 TRs, and 2 Opns (Supplementary Figure 1 and Table 1). It has been reported that ectopic expression of ORs can be due to incomplete or chimeric expression (Flegel et al., 2013). Thus, full-length primer sets were designed to confirm the expression of the entire coding region for all genes of interest. Following this approach, we were able to confirm full-length expression of 6 ORs from the initial screen (Olfr99, Olfr267, Olfr1366, Olfr57, Olfr78, and Olfr177). In addition, we also found full-length expression of Olfr16 and Olfr544, ORs that had not been included in our initial array cards but have known ligands, and in the case of Olfr544, known hepatic function (Griffin et al., 2009; Low and Mombaerts, 2017; Wu et al., 2017) (Figure 1A). Bitter TRs (T2Rs) have been shown to be activated by diverse ligands including physiologically relevant compounds such as bacterial-derived byproducts (Carey and Lee, 2019; Wu S. et al., 2019); thus, we limited our TR screen to confirm identity of the full-length bitter T2Rs resulting in the confirmation of 6 (Tas2r108, Tas2r126, Tas2r137, Tas2r135, Tas2r138, and Tas2r143; Figure 1B). Finally, we confirmed full transcript expression of one Opn, Opn3 (Figure 1C).

### Taste Receptors Are Localized to Hepatocytes and a Hepatocyte Cell Line

**FIGURE 2:**
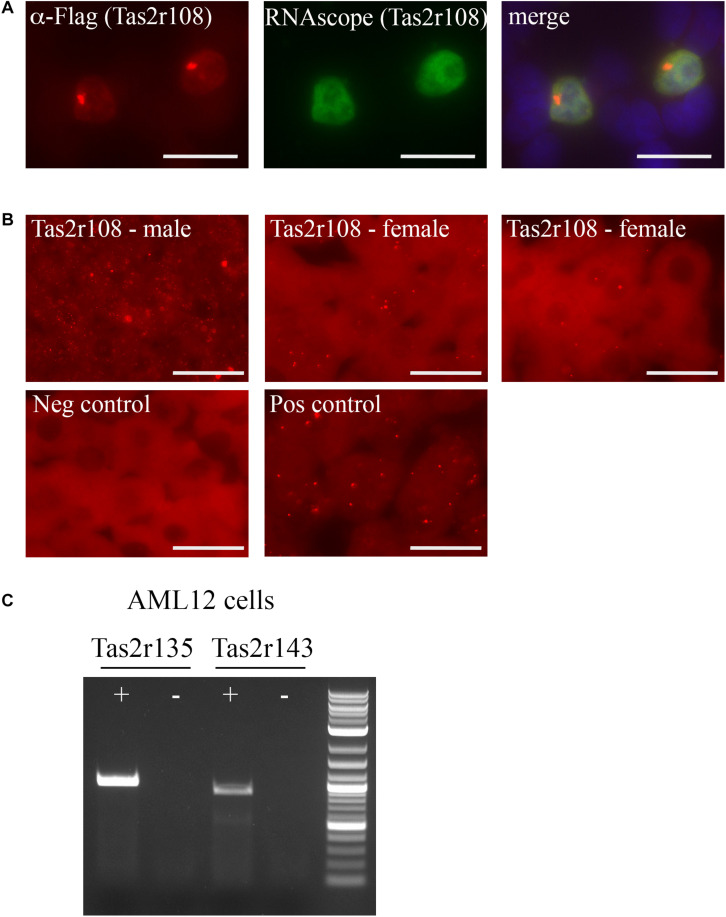
Tas2r108, Tas2r135, and Tas2r143 are expressed in hepatocytes and a hepatocyte cell line. **(A,B)** RNAscope was performed using an anti-sense probe designed against Tas2r108. **(A)** Immunofluorescence (red) and RNAscope (green) was performed on HEK293T cells overexpressing Flag-Tas2r108. DAPI (blue) indicates nuclei. The probe specifically detected only those cells expressing the construct, confirming probe reliability. Scale bar = 20 μm. **(B)** RNAscope was performed on both male and female liver sections for Tas2r108 (red – top row) with fluorescent puncta indicating positive signal and seen only within hepatocytes. The corresponding negative and positive (RNAscope for HS-PPIB) controls are shown below. Scale bar = 20 μm. **(C)** RT-PCR for full-length taste receptors was performed in murine hepatic AML12 cells with Tas2r135 and Tas2r143 detected in the cell line. += RT reaction, – = mock RT reaction.

Function of the sensory receptors is often dependent on the localization of the receptors, however, there are few antibodies available for these proteins, and the ones that do exist from commercially available sources are typically unreliable (Shepard and Pluznick, 2015). Thus, to glean potential function from these receptors, we utilized RNAscope, a method that relies on unique probes to localize individual mRNA strands in a tissue section. There is an existing commercially available probe for Tas2r108 and we confirmed its function using over-expressed Flag-Tas2r108 in HEK293T cells. The probe successfully detected only those cells that had been transfected with Flag-Tas2r108 as indicated by the colocalization with the anti-Flag staining (Figure 2A). With the reliable probe, we were able to confirm expression of this receptor in both male and female liver samples each displaying a variation in expression levels based on the number of RNAscope puncta that were observed (Figure 2B). In all samples, it appeared that Tas2r108 was confined to the hepatocytes, the parenchymal cells of the liver.

To identify additional hepatic bitter T2Rs, we turned to AML12 cells, a cultured murine hepatocyte cell line (Wu et al., 1994; Bell et al., 2008). Full length primer sets for all 6 TRs were used to screen for expression in the AML12 cells with both Tas2r135 and Tas2r143 exhibiting detectable expression (Figure 2C). Notably, we did not detect Tas2r108 suggesting that the AML12 cells do not completely mirror that of the *in vivo* expression pattern or that its level of expression in the cell line was too low to detect.

### Olfr57 Is Found in Hepatocytes

**FIGURE 3:**
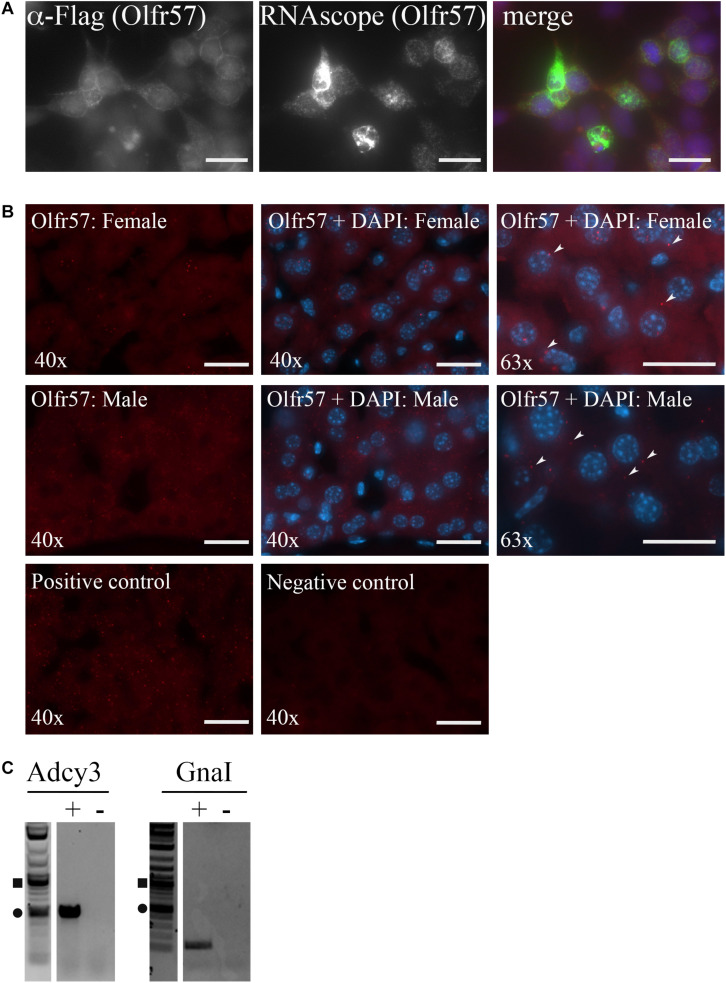
Olfactory receptors and downstream signaling machinery are localized within the liver. **(A,B)** RNAscope was performed using an anti-sense probe designed against Olfr57. **(A)** Immunofluorescence (left panel, red) and RNAscope (middle panel, green) was performed on HEK293T cells overexpressing Flag-Rho-Olfr57. DAPI (blue) indicates nuclei. The probe specifically detected only those cells expressing the construct confirming probe reliability. Scale bar = 20 μm. **(B)** RNAscope was performed on both male and female liver sections for Olfr57 (Cy5; red) with images taken at both 40 and 63×. DAPI staining to detect nuclei (blue) indicates that Olfr57 expression was mainly detected in hepatocytes, although some non-hepatic expression was also noted. Little to no fluorescent puncta were observed in the negative (neg) control and the positive control (HS-PPIB) shows a similar number of puncta. Arrow heads indicate areas of positive staining within hepatocytes. Scale bar = 20 μm. **(C)** RT-PCR on whole liver cDNA confirmed expression of Adcy3 that encodes for adenylyl cyclase 3 and GnaI that encodes for *G*_α *olfactory*_, the two downstream signaling proteins of the olfactory receptor signaling cascade. += RT reaction, – = mock RT reaction.

To begin to localize hepatic ORs, we generated a custom RNAscope probe for Olfr57, one of our highest expressing orphan ORs. The specificity of this probe was first tested on HEK293T cells overexpressing Flag-Rho-Olfr57 where we found that the probe could detect Olfr57 mRNA in only those cells expressing the construct (as indicated by the colocalization of the Olfr57 RNAscope and anti-Flag antibody staining seen in merge; Figure 3A). When RNAscope was performed in both male and female livers, we detected low expression of this transcript. Positive RNAscope puncta can be seen in the cytoplasm of hepatocytes (Figure 3B insets) with some potential expression noted in non-hepatic cells as well. While the puncta were not abundant, the staining was notable compared to the negative control (no probe added).

In the nose, ORs are known to couple to *G*_α *olfactory*_ leading to activation of adenylyl cyclase 3 (Buck and Axel, 1991; Buck, 2005). To determine if this signaling pathway is conserved in the murine liver, RT-PCR for these genes was performed on whole liver cDNA. Indeed, we did detect expression of both Adcy3 and GnaI (Figure 3C) implying that the hepatic ORs may couple to this pathway in the liver as well.

### Hepatic Olfactory Receptors Respond to Methylpyrazines and Terpenes

**FIGURE 4:**
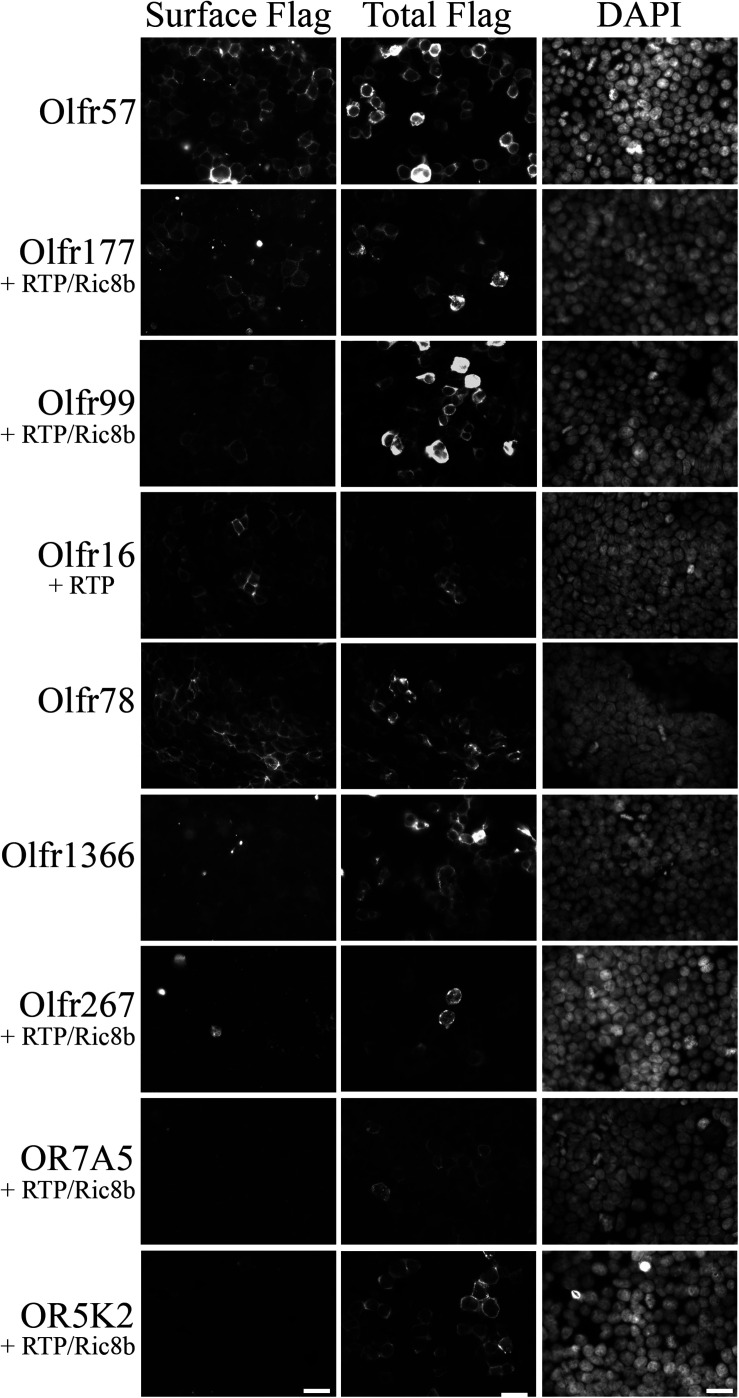
Functional surface expression was achieved for 5 murine olfactory receptors. Hepatic olfactory receptors were cloned into a vector containing several N-terminal tags: Lucy and Rho to optimize surface expression and Flag for detection purposes. Receptors were transfected into HEK293T cells either alone or in combination with trafficking proteins RTP1s and/or Ric8b, and then live-surface labeled with a polyclonal Flag antibody to detect the protein expressed on the cell surface (Surface Flag). Cells were then fixed, permeabilized, and stained with a monoclonal Flag antibody to detect all transfected cells (Total Flag). DAPI was used to visualize nuclear staining. Scale bar = 20 μm.

The expression of hepatic ORs suggests that these receptors are sensing physiological compounds within the liver. Unfortunately, to date, the majority of ORs remain orphan receptors with no known ligands. This is due, in large part, to the inability to successfully express the receptors in heterologous cell systems, a prerequisite for most deorphanization assays (Touhara et al., 1999; Katada et al., 2003; Zhuang and Matsunami, 2008; Shepard et al., 2013). To identify conditions that promote surface expression, the full-length OR products (Figure 1A) were cloned into an expression vector containing several N-terminal tags: a cleavable Lucy tag (Shepard et al., 2013) and the Rho tag (Krautwurst et al., 1998), both of which aid in OR trafficking, as well as a Flag tag for detection purposes. The cloned receptors were transiently transfected into HEK293T cells, either alone, or co-transfected with RTP1s, or a combination of RTP1s and Ric8b trafficking proteins, both of which have been reported to aid in OR trafficking (Von Dannecker et al., 2006; Fukutani et al., 2019). To screen for surface expression, live, non-permeabilized transfected cells were first surface labeled with a polyclonal Flag antibody to assess surface expression. Subsequent fixation, permeabilization, and probing with a monoclonal Flag antibody was used to detect total receptor expression (Figure 4). From these studies, we were able to identify surface-expression conditions for 5 out of 7 cloned murine ORs. Olfr57 and Olfr78 [as previously reported (Pluznick et al., 2013)] were able to reach the cell surface without the addition of any chaperone proteins, Olfr16 reached with the aid of RTP1s, and Olfr99 [also previously reported;(Shepard et al., 2013)] and Olfr177 required co-expression of both RTP1s and Ric8b. We were unable to achieve surface expression conditions for Olfr1366 or Olfr267.

Three ORs identified in our screen have known ligands (Olfr78, Olfr16, and Olfr544). Olfr78 and its human homolog, OR51E2 are known to respond to short-chain fatty acids produced by gut microbiota, β-ionone, and androstenone derivatives (Pluznick et al., 2013). Olfr16 and its human homolog, OR10J5, can be activated by acetophenone, α-cedrene, and lyral (von der Weid et al., 2015; Tong et al., 2017). Olfr544 is activated by azelaic acid (Abaffy et al., 2007; Kang et al., 2015; Thach et al., 2017). Olfr57, Olfr99, and Olfr177, on the other hand, are all orphan receptors. Having established functional expression for all three orphan receptors in HEK293T cells, we performed a cAMP-dependent dual luciferase reporter assay to identify ligands. Initially, each OR was tested against a suite of odorant mixes covering a wide odorant space (Pluznick et al., 2013) as well as physiologically relevant compounds. In addition, for those ORs with deorphanized “family members” according to the MOR nomenclature (Zhang and Firestein, 2002), these compounds were also screened as related ORs often respond to similar classes of odorants (refer to Supplementary Table 1 for a complete list of compounds screened). For the initial deorphanization assays, all compounds tested were screened at a minimum of two concentrations: 100–300 μM and 1–5 mM to cover a wide range of potential activation thresholds. While this screening method did not reveal any ligands for Olfr99, we successfully identified ligands for both Olfr177 and Olfr57.

**FIGURE 5:**
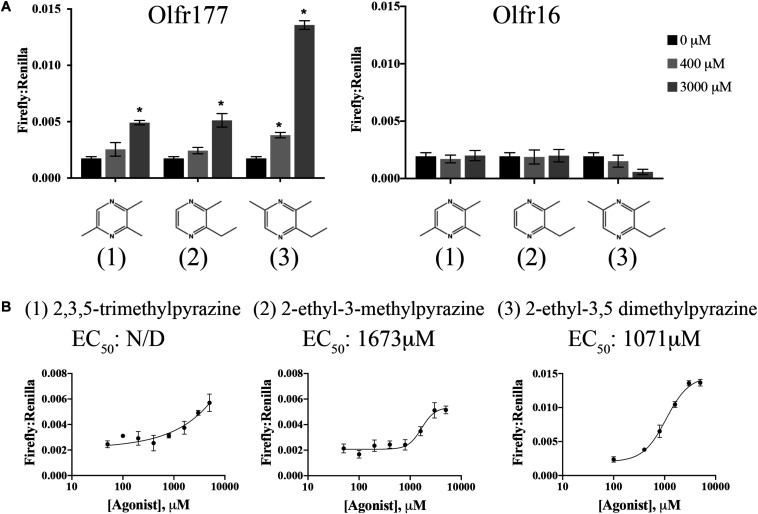
Olfr177 is activated by methylpyrazines. **(A)** Olfr177 was expressed in HEK293T cells under conditions of maximal surface expression (Figure 4) along with two luciferase reporter constructs, Firefly and Renilla. Cells were incubated with the identified compounds to elicit an activated cAMP response indicated by an increase in the ratio of Firefly to Renilla. A representative activation graph is shown for 2 concentrations of compounds (400 and 3,000 μM) with *p > 0.05 deemed significant activation over baseline. To confirm specificity, the same ligands were screened against Olfr16 (right) with no responses detected. The numbers shown in **(A)** correspond to the chemical names listed in **(B)**. **(B)** All three methylpyrazine agonists were tested at multiple concentrations to generate a dose-response curve and to calculate the EC_50_ values. Activation graphs are plotted as means ± SEM.

Use of the odorant mixes and other physiologically relevant compounds did not reveal any ligands for Olfr177. Olfr177 is a member of the MOR184 family (MOR184-7) and two related ORs, Olfr172 (MOR184-2), and Olfr173 (MOR184-3), are putative homologs for the human OR OR5K1, which is known to be activated by 2-ethyl-3,5-dimethylpyrazine (Wilkin Françoise et al., 2015). When this compound was tested on HEK293T cells expressing Olfr177, we detected a significant increase in the Firefly:Renilla ratio indicating that it can also activate Olfr177 with a calculated EC_50_ of 1,071 μM (Figures 5A,B). 2-ethyl-3,5-dimethylpyrazine is classified as a methylpyrazine and exploration of this chemical space identified an additional two ligands for Olfr177: 2-ethyl-3-methylpyrazine (EC_50_ = 1,673 μM) and 2,3,5-trimethylpyrazine (EC_50_ undetermined) (Figure 5). Activation of Olfr177 by these methylpyrazines was specific for this receptor; these compounds did not elicit a cAMP response when tested against Olfr16 (Figure 5A). Notably, unmodified pyrazine did not elicit OR activation.

**FIGURE 6:**
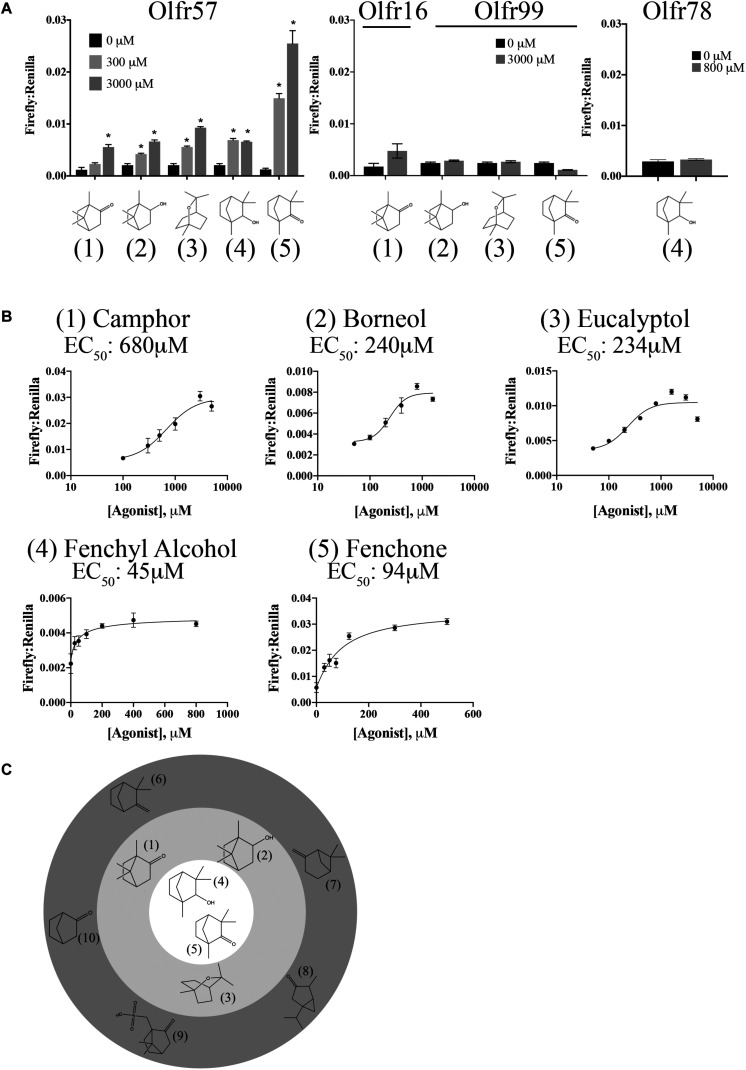
Olfr57 is activated by monoterpenes. **(A)** Olfr57 was expressed in HEK293T cells under conditions of maximal surface expression (Figure 4) along with two luciferase reporter constructs, Firefly and Renilla. Cells were incubated with the identified compounds to elicit an activated cAMP response indicated by an increase in the ratio of Firefly to Renilla. A representative activation graph is shown for 2 concentrations of compounds (300 and 3,000 μM) with *p > 0.05 deemed significant activation over baseline. To confirm specificity, the identified ligands were also screened against another OR (Olfr16, Olfr99, or Olfr78) at the highest dose. No responses were detected. The numbers shown in **(A)** correspond to the chemical names listed in **(B)**. **(B)** All 5 monoterpene agonists were tested at multiple concentrations to generate a dose-response curve and to calculate the EC_50_ values. Activation graphs are plotted as means ± SEM. **(C)** The chemical structures of all activators and some notable ‘non-activators’ are shown relative to their potency. The inner circle indicates the best activators as determined by their EC_50_ values, while those compounds in the middle ring represent the remaining activators. Those compounds that are structurally similar but did not elicit a response are shown in the outermost ring. (Agonists: 1 – camphor; 2 – borneol; 3 – eucalyptol; 4 – fenchyl alcohol; 5 – fenchone. Notable non-activators: 6 – camphene; 7 – pinene; 8 – thujone; 9 – camphorsulfonic acid; 10 – norcamphor).

As for Olfr177, the odorant mixes and other general compounds also did not activate Olfr57. Luckily, this receptor belongs to the MOR139 subfamily (alias MOR139-3) that contains one deorphanized OR, MOR139-1. MOR139-1 has been reported to respond to (1R)-(+)-camphor (Hiroaki Matsunami, 2010), which we determined can also weakly activate Olfr57 (EC_50_ = 680 μM; Figures 6A,B). Upon confirmation of camphor’s agonist activity, other structurally similar compounds were also tested leading to the discovery that (1R)-(-)-fenchone (EC_50_ = 94 μM), fenchyl alcohol (EC_50_ = 45 μM), borneol (EC_50_ = 240 μM), and eucalyptol (EC_50_ = 234 μM) are also activators of Olfr57 (Figures 6A,B). Activation was specific for Olfr57 as these compounds failed to activate Olfr16, Olfr99, or Olfr78 at their highest activating concentration (Figure 6A). Collectively, all of these ligands are bicyclic monoterpenes; notably, several structurally similar monoterpenes including camphene, norcamphor, thujone, pinene, and camphorsulfonic acid did not elicit a cAMP-mediated response from Olfr57 (Figure 6C), suggesting that a “boat” formation with an opposing oxygen is a required motif for Olfr57 ligand binding.

Both Olfr177 and Olfr57 have putative orthologs reported by NCBI: OR7A5 for Olfr57 and OR5K2 for Olfr177. Unfortunately, efforts to confirm conserved activation for these human ORs were unsuccessful as neither successfully trafficked to the cell surface (Figure 4).

## Discussion

Sensory receptors including ORs, TRs, and Opns have important physiological functions throughout the murine and human bodies with many of these GPCRs responding to naturally produced metabolites. While individual reports of several hepatic ORs have recently been described (Wu et al., 2017; Li et al., 2019), our data represent the first large-scale screen for these receptors in the liver. Using a custom-generated TaqMan Array screen along with molecular biology follow-up experiments, we confirmed expression of 7 ORs, 6 bitter T2Rs, and 1 non-visual Opn in murine liver. Notably, we uncovered localization profiles for several of these receptors and deorphanized two ORs, which respond to methylpyrazines (Olfr177) and monoterpenes (Olfr57). Together, our data suggest that the liver utilizes a variety of understudied sensory receptors to maintain homeostatic functions.

What are the physiological roles of these newly identified hepatic sensory receptors? Our data, coupled with other recent reports from the literature, shed some insight into the chemosensory functions of the liver. To date, there are many well-characterized ectopically expressed ORs, including Olfr78, which we also found in our screen (Pluznick et al., 2013). Recently, several reports have emerged that implicate ORs in steatosis and glucose metabolism. In cultured hepatocytes, murine Olfr43 has been shown to reduce hepatic lipid accumulation and adiposity while its human homolog (OR1A1) has been shown to reduce PPAR-γ expression (Wu et al., 2019). Another study found that stimulation of Olfr43 led to the secretion of glucose-like phosphoprotein-1 (GLP-1) in enteroendocrine cells, which in turn helps regulate blood glucose levels in a type-2 diabetic mouse model (Kim et al., 2017). In this study, we were able to confirm full-length expression of Olfr544 which had been previously found in the liver by Wu et al. (2017). This OR, which responds to Azelaic acid, can reduce adiposity and drive fuel-preference toward lipids in high-fat diet conditioned mice, and stimulate lipolysis in cultured adipocytes (Wu et al., 2017). Finally, Olfr734 and its hormone activator, Asprosin, has recently been shown to modulate gluconeogenesis and adiposity (Li et al., 2019). Indeed, a common theme amongst these ORs is their ability to contribute to lipid formation and utilization. While it remains to be seen whether any of our newly identified hepatic ORs (Olfr57, Olfr177, Olfr16, and Olfr99) contribute to similar processes, their ligand profiles suggest they might.

Olfr16 has several known ligands including lyral and α-cedrene, the latter of which promotes reduction in triglyceride, cholesterol, and free fatty acids along with lowered circulating levels of AST/ALT in treated mice (Tong et al., 2017). These findings are relevant to the human condition, as intracellular lipid accumulation was diminished in human HepG2 cells treated with this same agonist (Tong et al., 2017).

Using a cAMP-driven dual luciferase reporter assay, we were able to assign ligands to the previously orphaned ORs, Olfr57 and Olfr177. Compared to Olfr57, Olfr177 appears to be more narrowly tuned receptor, responding to several methylated pyrazines, though not to pyrazine in its non-methylated state. Notably, in the hepatocellular carcinoma line (HepG2), heme oxygenase activity was induced by 2-Ethyl-3,5-dimethylpyrazine (Parra, 2012), which we found to activate Olfr177. This odorant was also found to inhibit phosphodiesterase activity (Parra, 2012). While there are indications of physiological relevance for these methylpyrazines, the calculated EC_50_ values are considerably higher than is typical for ORs (>1 mM). While there are reports of several ectopically expressed ORs that are activated in the millimolar range (Pluznick et al., 2013; Shepard et al., 2016) it remains a distinct possibility that we have yet to identify the truly physiologically relevant ligands for Olfr177.

Olfr57 is a bit more of a broadly tuned receptor, responding to several ligands all in the class of monoterpenes including its strongest activators, Fenchyl Alcohol and (1R)-(-)-Fenchone. These terpenes are commonly found in analysis of several essential oils (Yan et al., 2013) and both camphor and fenchone have been shown to be metabolized, not only in the liver, but by the same microsomal enzyme CYP2A6 (Gyoubu and Miyazawa, 2007; Miyazawa and Gyoubu, 2007). Camphor, fenchone, fenchyl alcohol, and borneol (all activators of Olfr57) were also found to elicit antibiofilm and antihyphal properties (Manoharan et al., 2017). In a rat model of hepatotoxic liver injury, fennel oil [which contains both fenchone and camphor (Diao et al., 2014)], was protective against liver damage as indicated by reduced AST, ALT, ALP, and bilirubin (Ozbek et al., 2003). Lavender essential oils largely comprised of camphor and fenchone (45.25% total composition), were also found to possess antioxidant properties in an alloxan induced rat model of diabetes (Sebai et al., 2013), again raising the possibility that these compounds possess hepatoprotective properties. There have also been recent claims that monoterpenes may have anti-diabetic properties (Habtemariam, 2017) and may modulate cholesterol synthesis via the inhibition of HMG-CoA reductase (Clegg et al., 1982; Elson and Yu, 1994; Ren and Gould, 1994). Finally, it has been shown that several of these monoterpenes can be synthesized and released by fungi (Elke et al., 1999), raising the possibility that commensal fungi could be impacting liver function via Olfr57.

While the primary focus of this study was on the characterization of hepatic ORs, we did find several T2Rs and Opns as well. As their name implies, bitter T2Rs respond to notably bitter compounds and these receptors and their activators have been found throughout the body. Notably, bitter chemicals have been shown to alter food intake via modulation of ghrelin (Janssen et al., 2011), contribute to lipid metabolism (Cancello et al., 2020), and accelerate microorganism clearance in the ciliated airway (Shah et al., 2009). With respect to the liver, bile acids are extremely bitter substances (Sansome et al., 2020), leading to speculation that some of the identified T2Rs respond to these steroid acids that are produced by the liver. Using both RNAscope and hepatic AML12 cell, we localized a total of 3 bitter T2Rs to hepatocytes, the parenchymal cells of the liver (Tas2r108 and via RNAscope and Tas2r135 and Tas2r143 in AML12 cells). It should be noted that we were unable to confirm the expression of either Tas2r108 in AML12 cells, suggesting that these cells do not fully recapitulate *in vivo* conditions. Nevertheless, the localization of these receptors to the hepatocytes suggest that they may contribute to metabolism and xenobiotic processing. Indeed, ligands for Tas2r108 have been shown to improve glucose tolerance and reduce liver adiposity in a mouse model, and may also have therapeutic value for Polycystic Ovary Syndrome (Wu S. et al., 2019). Expression for this receptor was confirmed in both male and female livers, and while we did not detect any quantifiable sex differences in expression, we do note more Tas2r108 puncta in male livers. Given this receptor’s potential role in glucose tolerance and adiposity, we cannot rule out that it is more highly expressed in livers from male mice. While we have yet to explore the physiological function of these hepatic bitter T2Rs, we speculate that they serve as xenobiotic chemosensors that function in the regulation of metabolic processes. Clearly this hypothesis warrants further testing. While we limited our follow-up to the bitter T2Rs, we also identified several sweet T1Rs (T1Rs; Supplementary Figure 1). Notably, in a T1R2 whole-body knockout mouse line, these mice exhibited increased expression of genes associated with lipogenesis and decreased accumulation of hepatic triglycerides when compared to their wild-type littermates maintained on a high-fat/low-carbohydrate diet (Smith et al., 2016). This finding is particularly remarkable given that the KOs had nearly a twofold increase in caloric consumption, though mitigated by a slight, albeit significant, increase in energy expenditure (Smith et al., 2016). These findings raise the possibility of ectopic TRs having metabolic functions driven in part by the liver.

Finally, we were able to confirm the full-length expression of one non-visual Opn, Opn3. High expression of this receptor in cancerous tissues, including lung adenocarcinomas, correlates with poor prognosis and is linked to enhanced cellular proliferation and epithelial-to-mesenchymal transition (Xu et al., 2020). Indeed, blue light stimulation has been shown to stimulate wound healing (Buscone et al., 2017; Castellano-Pellicena et al., 2019; Ozdeslik et al., 2019) suggesting that Opn3 contributes to cell migration. With regards to liver function, a recent report has shown that it has a role in adiposity and insulin resistance (Sato et al., 2020) and a similar mechanism might be at play in the hepatocytes. How this light-sensing receptor becomes activated in the liver remains to be seen, and future studies using *in vivo* and *in vitro* models should be focused at addressing this open question.

Our discovery that the liver expresses several ORs and TRs, coupled with the liver’s role in nutrient sensing and detoxification, provide promising evidence that sensory receptor signaling contributes vital information to help steer fuel preferences and possibly protect against foreign bodies or cancerous growth. Further research will be required to determine the mechanism of downstream effects of these receptors in the liver. Though there is some evidence that monoterpenes are highly bioavailable through oral administration in humans (Zimmermann et al., 1995), understanding under what circumstances these odorants and tastants are likely to be present in circulation would also improve our understanding of how the liver may be responding to its environment.

## Data Availability Statement

The data generated in this study was deposited into the GEO (accession: GSE157843, https://www.ncbi.nlm.nih.gov/geo/query/acc.cgi?acc=GSE157843).

## Ethics Statement

The animal study was reviewed and approved by Georgetown University Animal Care and Use Committee, Georgetown University.

## Author Contributions

RK, MB, and BS conceived and designed the experiments. RK, LS, MB, DF, and BS performed the experiments. RK and BS analyzed the data. RK and BS wrote the manuscript. All authors contributed to the article and approved the submitted version.

## Conflict of Interest

The authors declare that the research was conducted in the absence of any commercial or financial relationships that could be construed as a potential conflict of interest.
