# Supplementary figures and images for: The Sensing Liver: Localization and Ligands for Hepatic Murine Olfactory and Taste Receptors
Source: Front Physiol. 2020 Oct 6;11:574082. doi: 10.3389/fphys.2020.574082 (PMC7573564; doi:10.3389/fphys.2020.574082)

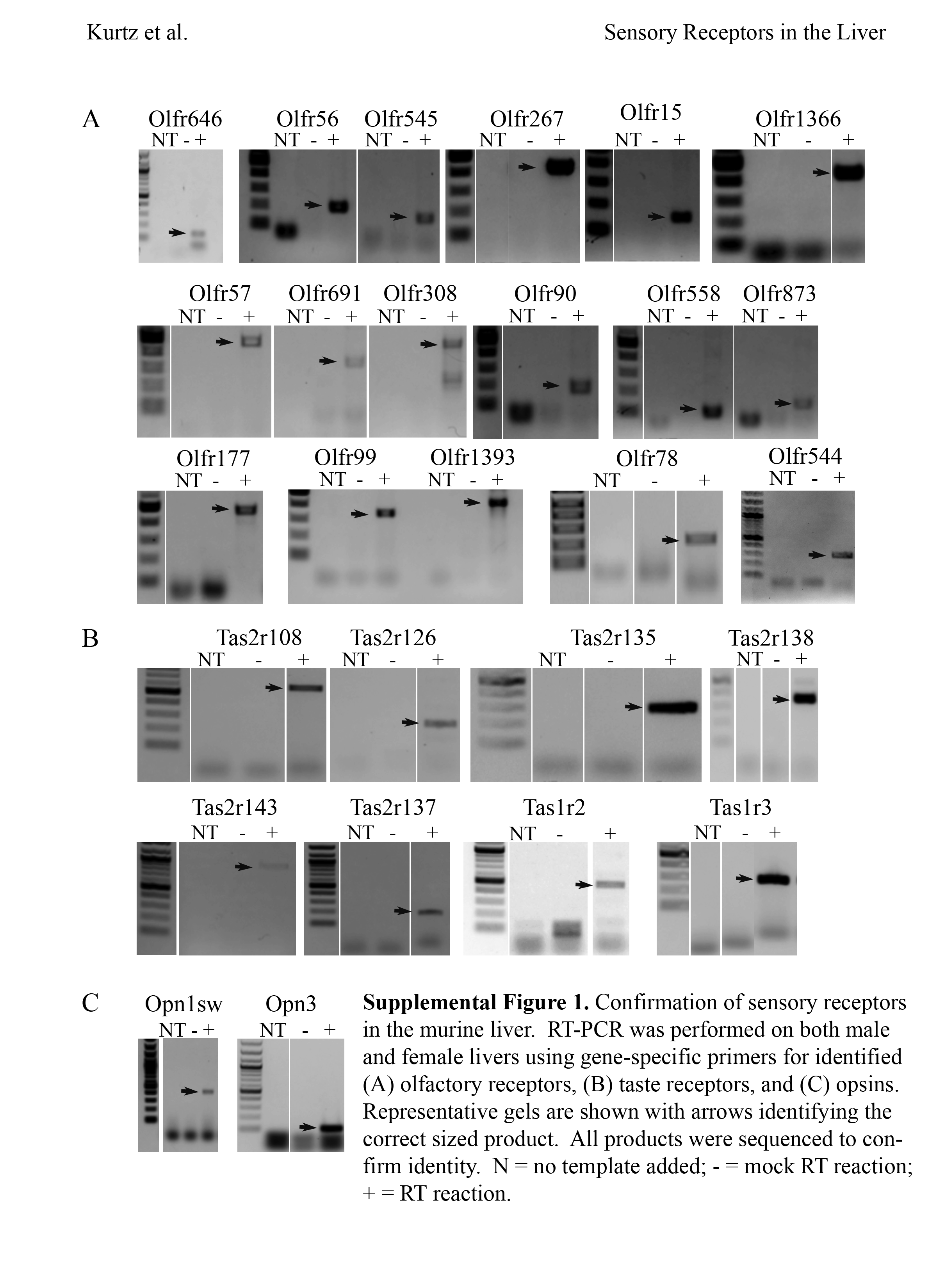

Supplement: Supplementary file 1 [file Image_1.tif]
