# Supplementary material for: The Sensing Liver: Localization and Ligands for Hepatic Murine Olfactory and Taste Receptors
Source: Front Physiol. 2020 Oct 6;11:574082. doi: 10.3389/fphys.2020.574082 (PMC7573564; doi:10.3389/fphys.2020.574082)
Supplement: Supplementary file 2 [file Table_1.pdf]

Supplemental Table 1: Custom TaqMan Array Cards	
**Gene Symbol**	**RefSeq**
**Bitter Taste Receptors (N=35)**	
Tas2r120	NM_207023.1
Tas2r109	NM_207017.1
Tas2r137	NM_001025385.1
Tas2r124	NM_207026.1
Tas2r108	NM_020502.1
Tas2r143	NM_001001452.1
Tas2r110	NM_199155.2
Tas2r135	NM_199159.1
Tas2r116	NM_053212.1
Tas2r129	NM_207029.1
Tas2r134	NM_199158.1
Tas2r126	NM_207028.1
Tas2r131	NM_207030.1
Tas2r105	NM_020501.1
Tas2r102	NM_199153.2
Tas2r104	NM_207011.1
Tas2r140	NM_021562.1
Tas2r125	NM_207027.1
Tas2r119	NM_020503.2
Tas2r136	NM_181276.1
Tas2r114	NM_207019.1
Tas2r106	NM_207016.1
Tas2r139	NM_181275.1
Tas2r118	NM_207022.1
Tas2r115	NM_207020.1
Tas2r103	NM_053211.1
Tas2r144	NM_001001453.1
Tas2r130	NM_199156.1
Tas2r113	NM_207018.1
Tas2r121	NM_207024.1
Tas2r123	NM_207025.1
Tas2r107	NM_199154.1
Tas2r122	NM_001039128.1
Tas2r117	NM_207021.1
Tas2r138	NM_001001451.1
**Taste Receptors for Umami and Sweet (N=3)**	
Tas1r3	NM_031872.2
Tas1r1	NM_031867.2
Tas1r2	NM_031873.1

Opsins (N=5)	
Opn4	NM_001128599.1;NM_013887.2
Opn1sw	NM_007538.3
Opn5	NM_181753.4
Opn1mw	NM_008106.2
Opn3	NM_010098.3
**Olfactory Receptors (N=44)**	
Olfr78	NM_001168503.1;NM_130866.4
Olfr322	NM_207693.1
Olfr732	NM_146665.2
Olfr558	NM_147093.3
Olfr435	NM_146653.1
Olfr13	NM_146652.1
Olfr658	NM_001171477.1
Olfr211	NM_146912.1
Olfr177	NM_146996.2
Olfr410	NM_146707.1
Olfr56	NM_010999.2
Olfr90	NM_146477.2
Olfr1352	NM_147071.2
Olfr166	NM_147068.1
Olfr418	NM_146651.2
Olfr355	NM_146625.1
Olfr314	NM_001011760.2
Olfr91	NM_182714.2
Olfr287	NM_001011780.1
Olfr288	NM_001011733.2
Olfr411	NM_146709.2
Olfr618	NM_147047.2
Olfr273	NM_146824.1
Olfr267	NM_146920.2
Olfr71	NM_019486.1
Olfr15	NM_008762.2
Olfr26	NM_146783.2
Olfr933	NM_146441.1
Olfr646	NM_147056.1
Olfr57	NM_147041.2
Olfr714	NM_147033.2
Olfr11	NM_146542.2
Olfr873	NM_146561.1
Olfr308	NM_146621.1
Olfr935	NM_146746.1

Olfr1366	NM_146283.2
Olfr1428	NM_146678.2
Olfr1392	NM_146470.2
Olfr1393; Olfr10	NM_146471.1;NM_206822.1
Olfr691	NM_147061.1
Olfr693	NM_146453.2
Olfr99	NM_146515.2
Olfr31	NM_147027.2
Olfr545	NM_146840.1
**Reference Gene**	
Gapdh	NM_008084.3;NM_001289726.1
