# Supplementary material for: The Sensing Liver: Localization and Ligands for Hepatic Murine Olfactory and Taste Receptors
Source: Front Physiol. 2020 Oct 6;11:574082. doi: 10.3389/fphys.2020.574082 (PMC7573564; doi:10.3389/fphys.2020.574082)
Supplement: Supplementary file 3 [file Table_2.pdf]

Supplemental Table 2: Chemical Screening					
**Compounds Tested[1]**	**Olfr16**	**Olfr57**	**Olfr99**	**Olfr177**	**CAS Number**
Odorant Mixes[2]					
BzB	o	o	o	o	124-19-6, 111-71-7, 110-62-3
BzC	o	o	o	o	6485-40-1, 97-53-0, 104-55-2
BzC-cinn	o	o	o	o	6485-40-1, 97-53-0
MA	o	o	o	o	628-63-7, 106-68-3, 98-86-2
OXLK	o	o	o	o	0431-03-08, 78-98-8, 64-19-7, 540-63-6, 78-93-3
THI-DI	o	o	o	o	1191-43-1, 540-63-6, 513-44-0, 1191-08-08, 4532-64-3
Physiologically Relevant Compounds					
Bile (Bovine/Ovine)	NT	NT	o	NT	8008-63-7
Chenodeoxycholic acid	NT	o	NT	NT	474-25-9
Cholic acid	NT	o	o	NT	81-25-4
D-Fructose	NT	o	NT	NT	7660-25-5
Deoxycholic acid	NT	o	o	NT	83-44-3
Fructose	NT	NT	o	NT	7660-25-5
Glucose	NT	o	o	NT	2280-44-6
Glucose-1-phosphate	NT	o	o	NT	59-56-3
Glycerol	NT	o	o	NT	56-81-5
Mouse urine – Female	NT	o	o	NT	n/a
Mouse urine – Male	NT	NT	o	NT	n/a
Sodium glycochendeoxycholate	NT	NT	o	NT	16564-43-5
Taurocholic acid	NT	o	o	NT	81-24-3
Compounds Eliciting a Physiological Response					
3-methylbutan-1-ol	NT	o	NT	NT	123-51-3
3-Octanone	NT	o	NT	NT	106-68-3
Acetic acid	NT	o	o	NT	64-19-7
Cyclohexanone	NT	o	NT	o	108-94-1
Decanoic acid	NT	o	o	NT	334-48-5
Diethyl disulfide	NT	o	NT	o	110-81-6
Dimethyl disulfide	NT	o	NT	o	624-92-0
Ethyl isobutyrate	NT	o	NT	o	97-62-1

Geraniol	NT	o	NT	o	106-24-1
N-nonanoic acid	NT	o	o	NT	112-05-0
Nonanal	NT	o	o	NT	124-19-6
Phenethylamine	NT	o	o	NT	64-04-0
Phenyl acetate	NT	o	NT	o	122-79-2
Potassium Acetate	NT	o	NT	NT	127-08-2
Phenyl Acetate	NT	o	NT	o	1191-16-8
Propionate	NT	o	NT	NT	72-03-7
Pyrimidine	NT	NT	NT	o	289-95-2
Pyruvaldehyde	NT	o	NT	NT	78-98-8
Quinine	NT	o	o	o	130-95-0
Sodium pyruvate	NT	o	o	NT	113-24-6
Odorants					
1-Acetyl-3-methylpiperidine	NT	o	o	o	4593-16-2
1-butanol	NT	o	o	o	71-36-3
1-Octanol	NT	o	o	NT	111-87-5
1,6-hexanedithiol	NT	o	NT	o	1191-43-1
2-cyclohexen-1-one	NT	o	o	NT	930-68-7
2-ethyl-3,5dimethylpyrazine	o	o	o	x	13925-07-0
2-ethyl-3-methylpyrazine	o	NT	NT	x	15707-23-0
2-Heptanone	NT	o	o	NT	110-43-0
2-hexanone	NT	o	NT	NT	591-78-6
2-pentanol	NT	o	NT	NT	6032-29-7
2-phenylethanol	NT	o	NT	o	60-12-8
2,3,5-trimethylpyrazine	o	NT	NT	x	14667-55-1
2,4 difluorobenzyl alcohol	NT	o	o	o	56456-47-4
2,5-dimethylphenol	NT	o	NT	o	95-87-4
2’-coumaranone	NT	o	NT	o	553-86-6
3-Nitrotoluene	NT	o	o	NT	99-08-1
Acetaldehyde	NT	o	o	NT	75-07-0
Acetophenone	o	o	NT	NT	98-86-2

Alpha-terpineol	o	o	NT	NT	98-55-5
Amyl acetate	NT	o	NT	NT	628-63-7
Benzaldehyde	NT	o	o	o	100-52-7
Benzene	NT	o	NT	o	71-43-2
benzyl acetate	NT	o	NT	o	140-11-4
Borneol	o	x	NT	NT	10385-78-1
Camphene	NT	o	NT	o	79-92-5
Camphor	o	x	o	o	76-22-2
Camphorquinone	NT	o	NT	NT	10373-78-1
Camphorsulfonic acid	NT	o	NT	NT	3144-16-9
Carvone	NT	o	o	o	99-49-0
Cintronellol	o	o	o	o	106-22-9
Coumarin	NT	o	NT	o	91-64-5
Cycloheptanol	NT	o	o	NT	502-41-0
Cyclohexanecarboxylic acid	NT	o	o	o	98-89-5
Cyclohexene oxide	NT	o	o	NT	286-20-4
Dihydrocarvone	NT	o	NT	o	5524-05-0
DL-Menthol	NT	o	o	NT	1490-04-6
Epsilon-Caprolactam	NT	o	o	NT	105-60-2
Epsilon-Caprolactone	NT	NT	o	NT	502-44-3
Ethyl maltol	NT	o	o	NT	4940-11-8
Ethyl vanillin	NT	o	o	o	121-32-4
Eucalyptol	o	x	NT	NT	470-82-6
Eugenol	NT	o	o	o	97-53-0
Fenchone	o	x	o	o	1195-79-5
Fenchyl alcohol	o	x	NT	NT	2217-02-9
Heptaldehyde	NT	o	o	NT	111-71-7
L-carvone	NT	o	o	NT	6485-40-1
Linalool	NT	o	NT	o	78-70-6
Methyl hexanoate	NT	o	o	NT	106-70-7
Methyl salicylate	NT	o	NT	o	119-36-8

Norcamphor	NT	o	NT	NT	497-38-1
Octan-3-ol	NT	o	NT	NT	22658-92-0
Octan-3-one	NT	o	NT	NT	106-68-3
Octanoic Acid	NT	o	o	o	124-07-2
Octanol	NT	o	o	NT	111-87-5
Phenethyl alcohol	NT	o	o	NT	60-12-8
Pinene	o	x	o	o	67762-73-6
Pyrazine	NT	NT	NT	x	290-37-9
Pyridine	NT	NT	NT	o	110-86-1
Sodium metaperiodate	NT	o	NT	NT	7790-28-5
Thujone	NT	o	NT	NT	546-80-5
Thymol	NT	o	NT	o	89-83-8
Valeraldehyde	NT	NT	o	NT	110-62-3
Whiskey Lactone	NT	o	o	NT	39212-23-2

[1]All compounds tested compounds were screened at a minimum of two doses (100-300μm and 1-5mM)
[2]Composition of chemical mixes included in Materials and Methods
NT = Not Tested
0 = No response
X = Response detected
