# Supplementary material for: The Sensing Liver: Localization and Ligands for Hepatic Murine Olfactory and Taste Receptors
Source: Front Physiol. 2020 Oct 6;11:574082. doi: 10.3389/fphys.2020.574082 (PMC7573564; doi:10.3389/fphys.2020.574082)
Supplement: Supplementary file 4 [file Table_3.pdf]

Supplemental Table 3: Gene-specific primers

Murine OR	Forward Primer	Reverse Primer	Product length (bp)
Olfr10	CCTGGTAACCCTGTTGTGGT	GGTGGTAAAGGGCCTGCAAT	181
Olfr1351	TGAACACAAGACGCTGTGGA	AGAAGGTAAGTCCGTTAGTCTGTT	548
Olfr1366	CTGGTGGCTTTCTCTTGGGT	TCAATACGGGGGCTACAACC	427
Olfr1386	AAGAGCAGTTATTCTGAAAGCAAGG	CTCTTAGTCTGTGCCTCTCCC	150
Olfr1428	TCTTCCTCAAGTTGGTCTGTGAA	AGGAGCCTAGAATGAGCATGG	105
Olfr15	GTATGTGGCAGTTTGTCGGC	ATGACGCTTACTGGGACCAC	276
Olfr1507	CACCATCAAGCTGCCCTACT	GGGTTCAGCAGAGGGGTTATG	366
Olfr1508	CACCATCAAGCTGCCCTACT	TCAGTAGAGGGGTAACAGCAG	362
Olfr151	TGCCGTAGGACTCATCGGTT	CAGTGTTTTGTCCAGTGCGG	466
Olfr155	GTGCCGTGCAGATGTTTCTC	TCATTGCACCACTCTTCCCT	730
Olfr16	TGGTCATTGTCCCACGGATG	CAAGGCACACATTCCCTTGC	206
Olfr166	CTTCTCTCTGGCTTTGTGTGG	AAGGACTCTTGTCATGGCCC	814
Olfr177	CCACAGGAGCCTACATAGCAG	TTTTCTTCATTGCTCTTTTCATCAC	489
Olfr267	TCACTGTCCTGGATTCACGC	GGGAACTCACGCAAGCTAGT	432
Olfr308	AGCTGCAGTCCCAAAAGCTC	CAGATGGCAAGGTAGCGGTC	156
Olfr418	AACCCCTTGAGGTACTCGGT	TAGGCAATGGAGGCACAACC	392
Olfr43	ACTTCATGATAGGCTTGGCAAAT	GTCAGGGGCCGGAAATACAT	484
Olfr544	CCTTATTGTCTTTGACTGCAACAT	TCGGTTGAAGATGCGAACAG	304
Olfr545	CCATTGTCTACCGTGTGGCT	AGGAATGACCTTACCAGGTGC	148
Olfr554	GCTGTGGTAAGGGTAGAGCG	CAAGGAAGATGCCCGACTCA	221
Olfr558	AGGCTGTCCTCATCAACCAAA	TGGAGTCGGTGGTCGTGTAT	169
Olfr56	CCTTTTCGACACCCTGCTCT	AACATTGCTGCCCCGTAGAA	192
Olfr57	AGCACAAGACGCTGTGGATT	GCTGTCTTTATGTCTTTGTTCCTCA	491
Olfr6	TCACTGGGTCACTGCACAAG	TGGATAGCGAAGAGGCCAAC	248
Olfr646	CTCACCCATCGCTTTGGTCA	GCCGACTCCGAATCTCCTTG	133
Olfr691	TGTGCTAGTTGGCATCCCTG	ATGGGGGACGATGTTGGTTC	493
Olfr73	CTGTGCCATGCTGGTATTGG	ACAGATGTGTCAGAGCGTGA	162
Olfr78	AAGCGACTGGCTTTCTGTCA	GCCTTAGCTCGCTCAGACTT	221
Olfr873	TCGCCTCTGTGGCTCTTTAG	TAAGGAGTTGTGCAGGGTCAC	143
Olfr90	TCCTGGGATGCTTTGTCCAG	AACTACTGACTCCACTAGGCCA	194
Olfr904	CTGCACCAGCACCTATGTCA	CAAAATTTCCATCTGCTCAGGGT	369
Olfr918	GCTGACTGGAAATGGCTCCT	ATACATGAATGCCCCTGACCC	775
Olfr99	CACTGCGCTACTCAGCTCTC	GAACTGCACGTGGACAATGCT	343
**Murine TR**	**Forward Primer**	**Reverse Primer**	**Product length (bp)**
Tas1r1	CCTTTCAGGGGCATGCAGTTA	GCTGCCCGTAGTCACCATAG	766

Tas1r2	CTACAACCTCATGCAGGCCA	CTCCTGGAAGGCAATGCAGA	514
Tas1r3	GAGGAACATGTGATGGGGCA	CCCCGAAGGTAGCTGCATAG	968
Tas2r106	GCACAGAAATGTTTCCTGGCA	GTCACTCTGACGTCCTTGTCT	453
Tas2r108	AGTCGCAGAATTGCCTCTCC	GCCTCATAGCACCCATGTG	577
Tas2r126	TCCTCTTCAGTTTGGGCACC	CGGACACCAAGATAGAGCCC	285
Tas2r135	GAGTGGCCATCAACCTTGGA	GCAGAACTGAGTACCAGCGT	288
Tas2r137	GCCCACAAAAGAGCCATGAG	TGGGAGCATGGCCACAAAT	219
Tas2r138	AGCTTTCCTGGTTTCCTCGG	GGAGGAACCTTGTGGACTGG	366
Tas2r143	AGAGTGGATGAGGAACCGGA	GCCATGGTATGTGCCTGAGT	585
**Murine OPN**	**Forward Primer**	**Reverse Primer**	**Product length (bp)**
OPN1SW	CATGCACTGATGGTGGTCCT	GGGATGGTGACAAGCCGTAA	419
OPN3	GTACTCGGGGAACCGTAGTG	AACGTTCATAGGCCAGCACA	410
OPN4	GCTGTCTTTGGCATCACTTCC	ACGAGTGCCGTTCGTCTTT	119
**OR Signaling**	**Forward Primer**	**Reverse Primer**	**Product length (bp)**
GNA1	AGGATCCTGCACGTCAATGG	CGCCACGTAAATGATCGCAG	519
Adcy3	CCTTGCCCAACTTTGCTGAC	TCACAAAGATGGGACCTCGC	543
