# Supplementary material for: The Sensing Liver: Localization and Ligands for Hepatic Murine Olfactory and Taste Receptors
Source: Front Physiol. 2020 Oct 6;11:574082. doi: 10.3389/fphys.2020.574082 (PMC7573564; doi:10.3389/fphys.2020.574082)
Supplement: Supplementary file 5 [file Table_4.pdf]

Supplementary Table 4: Full Length Primers			
**Murine OR**	**Forward Primer**	**Reverse Primer**	**Product length (bp)**
Olfr90	ATGGTCAACCAGAGCTCCCC	CAGCCGGGCTTCACTTCC	932
Olfr78	ATGAGTTCCTGCAACTTCACCC	CACGTGTTTCCCCCAGCTTC	962
Olfr308	ATGATCACAGAGAATTGGACTCT	TCACTTTCTCTTCCATTTCTTCCCT	927
Olfr1366	ATGGAACTTTACTTCTCTCTATATCTC	TCACTTGGAACATATTTTGCTC	987
Olfr691	ATGATTCATAGCAACATCACCCC	TCATCCCAAAGGTGAAGAACA	969
Olfr558	ATGGTGGGCTTCAATAGCAA	CTAGTGATCTGAAGTGTGTGTGG	954
Olfr57	ATGGAAACAGGAAATGACACTCA	TTATGAAGGTGAATCCATTGATCTGC	960
Olfr646	ATGAGCGAAAGTCTCCCAGTC	TCATACTAAGTCCTTCCCCAAGTG	939
Olfr15	ATGGAGGTGGACAGCAACAGCT	TCAGCTGGCTCCTCTTCCTTTC	939
Olfr177	ATGACTGAGGACAACTACTCCT	TTATAATCTTTTCTTCATTGCTCTTTT	930
Olfr545	ATGTTGGGTTGGAGCAATGG	TCAGGGGCCTGCACTCAG	951
Olfr873	ATGTTCATTTTCAAAAGCTGTTCA	TTACAACATTCTTCTGTGAAGTCTCT	960
Olfr56	ATGGGAATATGGCTGAACGAATC	TCAATGTTGGCTGCCAACCC	948
Olfr267	CTTTCTCCAACAAGACGGGCGTGGTGGAATTCATGCCAGGAGAAAATGTCACTGTTTGGAGC	GGCGCAGAACTGGTAGGTATGGAAGATCCCTCGAGTCACACACTTGGTGCCAAGGGCAG	939
Olfr99	CTTTCTCCAACAAGACGGGCGTGGTGGAATTCATGAACTGCAGTCAGGCT	GGCGCAGAACTGGTAGGTATGGAAGATCCCTCGAGCTACTCCATGTTTAGGATTCTCCTC	915
Olfr16	ATGCAGAGAAATAACTTCACTGA	TTAAGAAGTGTTTCTGCCCA	930
**Murine TR**	**Forward Primer**	**Reverse Primer**	**Product length (bp)**
Tas2r106	ATCTGTAGAGATGCTGACTGTAGCAGAAGG	CTACCATGTCACTCTGACGTCCTTGTC	937
Tas2r108	ATGCTCTGGGAACTGTATGT	CTACTTGTAGAAACAGAAAATCTTCT	894
Tas2r126	ATGCTACCAACATTATCAGTTTTCT	CTAGGCCACCCAGAATCCCC	927

Tas2r135	ATTCGAGAGACTTCTTAGAGCAA CAAACTACCCTTC	TCAGCAGCAGCCCCTCTTTATCAC C	1002
Tas2r137	ATGAGATTTATGAACAGAACAAG CA	TTATGAAGCAGAGGGTCCCT	1002
Tas2r138	ATGCTGAGTCTGACTCCTGTC	TCAGAGTGTCCTGGGAGGA	996
Tas2r143	ATGCCCTCCACACCCACATT	CTAAAACCTCATCTTCAGGGCCT	882
Tas1r1	ATG CTT TTC TGG GCA GCT CAC CTG C	T CAG GTA GTG CCG CAG CGC	2528
Tas1r2	ATG GGA CCC CAG GCG AGG	CTA GCT CTT CCT CAT CGT GTA GCC CTG AAT	2531
Tas1r3	ATG CCA GCT TTG GCT ATC ATG GGT CTC	TCA TTC ATT GTG TCC CTG AGC TGC CT	2576
**Murine OPN**	**Forward Primer**	**Reverse Primer**	**Product length (bp)**
OPN1SW	ATGTCAGGAGAGGATGACTTTTA CC	TCAGTGAGGGCCAACTTTGCT	1041
OPN3	ATGTACTCGGGGAACCGTAG	TTATAGAGGACGCACTTGGATGA	1203
